# Visceral and subcutaneous adipose stem cells modulate colorectal cancer cell progression: direct and indirect contact distinctly accelerate tumor aggressiveness

**DOI:** 10.1186/s10020-026-01464-x

**Published:** 2026-03-23

**Authors:** Mikołaj Domagalski, Dorota Nowak, Piotr Schmidt, Katarzyna Pietraszek-Gremplewicz

**Affiliations:** Department of Cell Pathology, Faculty of Biotechnology, University of Wroclaw, Joliot-Curie 14a, 50-383 Wroclaw, Poland

**Keywords:** Adipose stem cells, Colon cancer, Tumor microenvironment, Direct co-culture, Indirect co-culture, Spheroid models

## Abstract

**Background:**

Adipose tissue is increasingly recognized as an important component of the tumor microenvironment of colorectal cancer (CRC) and actively contributes to the progression of the disease. Adipose stem cells (ASCs), one of its key constituents, can interact with cancer cells and contribute to tumorigenic processes. However, there is a poor understanding of the underlying basis of ASC-mediated support in the progression of CRC.

**Methodology:**

In this study, we employed direct and indirect co-culture models to investigate interactions between ASCs and colorectal cancer cells. The study was performed using human visceral ASCs (V-ASCs) and subcutaneous ASCs (S-ASCs), along with three colorectal cancer cell lines. The analyses primarily focused on the characteristics of CRC cell progression in 2D and 3D conditions. Cell proliferation and migration after indirect co-culture were assessed using video microscopy, XTT assay, wound healing, and spontaneous migration assay. Corresponding measurements for direct co-culture were performed using high-throughput confocal microscopy. Changes in the epithelial–mesenchymal transition (EMT) such as the phenotype and stemness features were evaluated by confocal microscopy imaging, while gene and protein expression were analyzed using qRT-PCR and Western blotting. Additional analyses were conducted using cells cultured in spheroid models with both indirect and direct cell–cell interaction to assess the spheroid formation capacity, phenotypic characteristics, and the ability of cells to migrate out of the spheroids.

**Results:**

The results demonstrate that paracrine interaction with ASCs results in increased migration and proliferation of CRC cells accompanied by EMT-related transcriptional and phenotypic changes and reduced levels of stemness-associated molecules. Notably, direct contact with ASCs potentiated these effects, which suggests more aggressive behavior of the CRC cells. The spheroid assays showed increases in spheroid formation and dispersal capacity of CRC cells with ASCs present under direct co-culture conditions. The findings indicate that both S-ASCs and V-ASCs were associated with comparable changes in CRC cell behavior, despite originating from distinct fat depots.

**Conclusions:**

The results suggest a potential role of ASCs in modulating the plasticity of CRC cells and specific aspects of aggressive behavior, including increased growth and motility, as well as an association with loss of stemness features through distinct interactions that affect tumor progression. This study indicates that targeting physical interactions may be a relevant complement to strategies focused on paracrine signaling within the tumor microenvironment in the development of new therapeutic approaches.

**Graphical Abstract:**

Green upward arrows indicate an increase, red downward arrows indicate a decrease, while blue bidirectional arrows represent ambiguous or inconsistent changes.

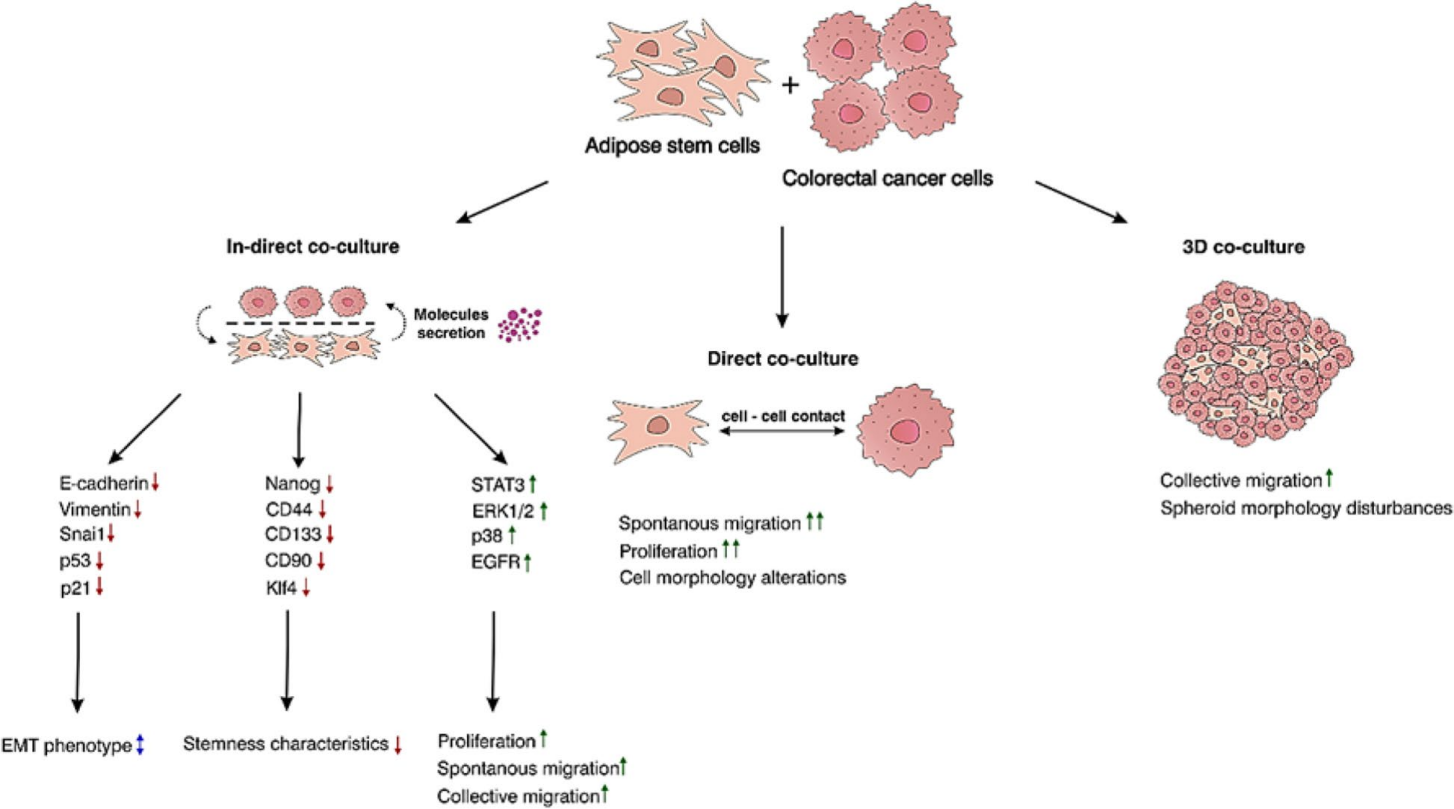

## Background

The tumor microenvironment (TME) of colorectal cancer (CRC) is composed of CRC cells and a spectrum of nontumor cells that are surrounded by extracellular matrix components (Domagalski et al. 2025). These elements form an intricate communication network that is governed by numerous secreted molecules (cytokines, growth factors, and adipokines) and by direct cell–cell contacts between tumor and non-tumor cells that modulate cancer behavior(Visser and Joyce 2023). Adipose tissue in particular has emerged as a pivotal element of the TME. Its primary constituents, adipocytes and adipose tissue stem cells (ASCs), have been found to be active TME components that are capable of influencing the growth and progression of tumors through both paracrine signaling and physical interactions with cancer cells (Domagalski et al. 2025; Visser and Joyce 2023). Despite the growing recognition of adipocytes significance in the TME, there is still very limited knowledge about how ASCs influence the biology of CRC cells.

ASCs are multipotent adherent cells that are present in adipose tissue and contribute substantially to the adipose secretome (Guillaume et al. 2022a; Xue et al. 2022a). Preclinical studies have implicated ASCs in inducing epithelial–mesenchymal transition (EMT) and promoting shifts in the molecular subtypes of CRC toward a mesenchymal phenotype (Xue et al. 2022a; Di Franco et al. 2021). Beyond EMT and stemness, ASCs may also influence the metabolism and resistance of CRC cells through pathways such as the TGF‑β1/SMAD3/ANGPTL4 pathway, thereby contributing to metastatic seeding (Zhu et al. 2024). ASCs have also been proposed as an element in the modulation of CRC cell stemness that increases sphere-formation capacity and supports metastatic traits in CRC and other types of tumors (Di Franco et al. 2021; Wei et al. 2015).

In CRC specifically, ASCs have been shown to secrete IL-6 and HGF, which expand the metastatic CRC stem-cell compartment (CD44v6 +) and promote mesenchymal characteristics (Di Franco et al. 2021). Adipose stem cells are present in both visceral fat depots (as V-ASCs) and subcutaneous fat (as S-ASCs). While they share typical MSC-like characteristics, they also differ in morphology, secretory profiles, and differentiation potential (Ritter et al. 2015, 2019). Visceral ASCs secrete higher levels of cytokines such as IL‑6, IL‑8, and TNFα than S-ASCs and exhibit altered migratory, oxidative‑stress, and differentiation characteristics (Ritter et al. 2019; Sriram et al. 2019). Recent studies suggest a correlation between the origin of depot-specific adipocytes and differential tumor progression, with the levels of subcutaneous or visceral depots being differentially associated with tumor incidence (Nimri et al. 2019). This functional heterogeneity provides a rationale for directly comparing V‑ASCs and S‑ASCs in the context of CRC progression.

Several important questions remain regarding the contribution of ASCs to CRC biology. It is not yet established whether ASC-driven effects on CRC are mediated by mainly paracrine signaling or whether they require direct cell–cell contact to further modulate cancer-cell behavior. The degree of functional divergence between V‑ASCs and S‑ASCs is insufficiently defined. Furthermore, it is still unclear what the relevance of ASC-induced changes is in three-dimensional models, which provide a better reflection of tumor architecture and intercellular dynamics. Addressing these gaps is essential for further understanding of the mechanisms through which adipose niches influence CRC progression.

We used indirect Transwell co-culture and direct co-culture as in-vitro approaches to clarify the interactions between CRC cells and ASCs and investigated the effect of V-ASCs and S-ASCs on CRC cell lines and direct spheroid co-culture. Moreover, we examined the precise potential differences resulting from different contact between ASCs and CRC cells (direct or indirect) in relation to CRC characteristics. We quantified canonical EMT markers, stemness features, migration, invasion, and proliferation abilities. Importantly, we directly compared the impact of indirect versus direct ASC–CRC interactions on spheroid biology by measuring the size, shape, and invasive outgrowth, as well as the spatial localization of CRC cells and ASCs within heterotypic spheroids. These measures were used to test the how ASC-driven programs (EMT, stemness, and invasiveness) affect the phenotypic plasticity of CRC cells. Furthermore, the use of heterotypic spheroids increases translational relevance by recapitulating tumor–stroma spatial organization, gradients of secreted factors, and cell–cell contacts that are not captured in 2D culture.

The results demonstrate that ASCs derived from two distinct fat depots promote pro-tumorigenic features of CRC cells under both direct and indirect co-culture conditions. Notably, CRC cells grown in direct contact with ASCs acquired a more aggressive phenotype and exhibited a characteristic EMT-like morphology. CRC cells maintained under indirect co-culture conditions were also characterized by reduced expression of stemness-associated markers and altered expression of EMT-related genes. In the 3D spheroid model, CRC–ASC co-cultures displayed pronounced alterations in spheroid morphology and organization both during and after formation, which were accompanied by more invasive properties in heterotypic spheroids composed of ASCs and CRC cells. The findings shed light on the role of adipose stem cells within the CRC microenvironment and suggest that their impact on CRC progression may depend on the fat depot of origin, which underscores the importance of direct cell–cell interactions.

## Materials and methods

### Cell culture

Human adipose-derived stem cells isolated from the visceral adipose depot of a 29-year-old female (InnoProt, Derio, Spain; catalog no. P10763; lot no. 22044) were characterized as CD90⁺, CD73⁺, CD105⁺, CD44⁺, CD13⁺, CD34⁻, CD45⁻, CD14⁻, CD19⁻, HLA-DR⁻. Human subcutaneous adipose-derived stem cells, obtained from a 42-year-old female (Lonza, Basel, Switzerland; catalog no. PT-5006; batch no. 22TL018258), were characterized as CD13⁺, CD29⁺, CD44⁺, CD73⁺, CD90⁺, CD105⁺, CD166⁺, CD14⁺, CD31⁺, CD45⁺, CD34⁺. Both cell types were cultured at passages 1–7 in DMEM (1 g/L glucose, 3.7 g/L NaHCO₃) at IITD PAN, Wrocław, Poland. The human CRC cell lines LS180 (RRID:CVCL_0397; Deutsche Krebsforschungszentrum, Heidelberg, Germany), HCT116 (RRID:CVCL_0291; European Collection of Cell Cultures, Salisbury), and LoVo (RRID:CVCL_0399; European Collection of Cell Cultures, Salisbury) were cultured in MEM-α (Sigma), McCoy’s 5 A (Sigma, Steinheim, Germany), and Ham’s Nutrient Mixture F12 (Sigma), respectively. All the media were supplemented with 10% fetal bovine serum (Gibco, Paisley, UK), 2 mM glutamine (Sigma), and an antibiotic/antimycotic cocktail (10 000 U/mL penicillin, 10 mg/mL streptomycin, and 25 µg/mL amphotericin B) (Gibco). All experiments were performed with mycoplasma-free cells and all used human cell lines have been authenticated. Cells were maintained in a humidified atmosphere at 37 °C with 5% CO₂ and passaged using a 0.25% trypsin/0.05% EDTA solution (IITD PAN).

### Co‑culture conditions

**Fig. 1:**
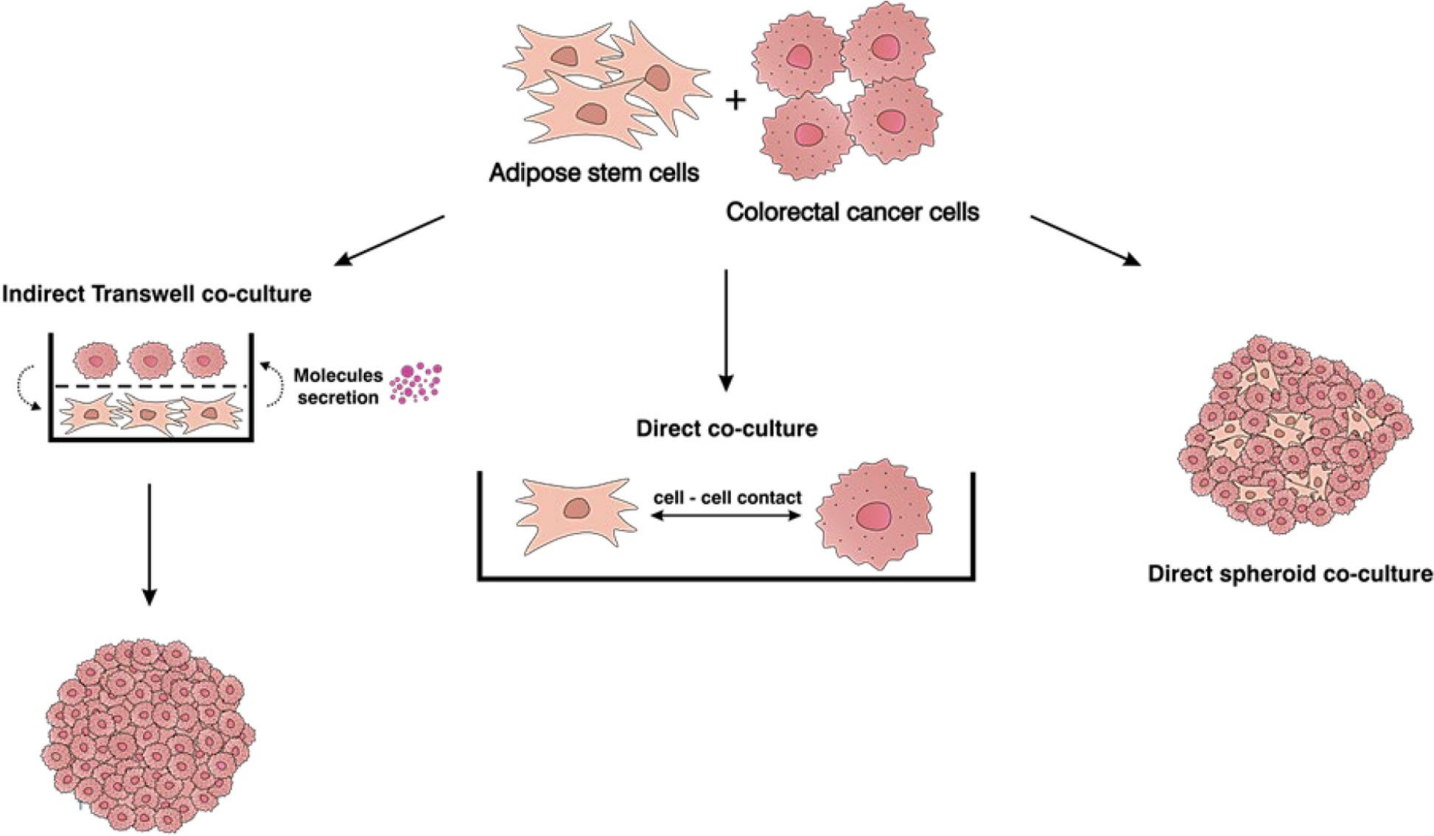
Schematic presentation of co-culture and spheroid models applied in the study. The model includes indirect co-culture of CRC cells and ASCs and further spheroid formation, direct co-culture, and spheroid direct co-culture

Indirect co-culture was performed using Transwell™ inserts (0.4 μm pores, Falcon) and 6-well plates (Fig. 1). After 24 h from ASCs (35,000 cells) seeding at the bottom of the plate (Corning, NY, USA), cancer cells (LS180: 75,000; HCT116: 25,000; LoVo: 80,000) were added to the inserts placed above the wells. Control cancer cells were cultured without ASCs (LS180: 150,000; HCT116: 50,000; LoVo: 160,000), with half of the medium replaced every 3 days.

In direct co-culture, ASCs (1000 cells/well) were seeded into a 96-well culture plate, and after 24 h, CRC cells were added at a 2.5-fold higher density (Fig. 1). Cells were maintained under co-culture conditions for the required time. For live-cell imaging, ASCs and CRC cells were stained with Cell Tracking Red Dye Kit (Abcam, Cambridge, UK) and Cytopainter (Abcam).

To establish direct spheroid co-cultures, solutions containing ASCs and CRC cells (LS180, HCT116, or LoVo) were prepared, with CRC cells added at 2.5-fold higher density than ASCs (Fig. 1). Spheroids were generated using the hanging-drop protocol described in the spheroid formation section. Cells were maintained under co-culture conditions for the indicated duration. For live-cell imaging, ASCs and CRC cells were stained with the Cell Tracking Red Dye Kit (Abcam, Cambridge, UK) and Cytopainter (Abcam). All co-culture experiments were performed in DMEM (1 g/L glucose, 3.7 g/L NaHCO₃) were supplemented with 10% fetal bovine serum (Gibco, Paisley, UK), 2 mM glutamine (Sigma), and an antibiotic/antimycotic cocktail (10 000 U/mL penicillin, 10 mg/mL streptomycin, and 25 µg/mL amphotericin B) (Gibco).

### Proliferation examination

CRC cell proliferation in indirect co-culture was assessed using the Cell Proliferation Kit II (XTT, Roche, Basel, Switzerland). For the XTT assay, 5,000 CRC cells were seeded in 96-well plates in a 1:1 mix of fresh and medium collected from co-culture. Proliferation was calculated as T_24_ absorbance normalized to T_0_ and compared to control conditions. In parallel, the proliferation rate was also assessed using the IncuCyte® Live-Cell Analysis System (Sartorius, Goettingen, Germany), where CRC cells seeded in IncuCyte ImageLock 96-well plates (Sartorius) were imaged every 2 h at 10 × magnification. Proliferation was quantified as the area covered by cells at 24 h, normalized to T_0_. Experiments were conducted in triplicate with three to four technical replicates per condition.

For direct proliferation assay ASCs were seeded into the wells of a 96-well plate (Revvity, Waltham, Massachusetts, USA) and incubated for 24 h. Next, CRC cells were seeded onto ASCs or into empty wells (control cells), and the plate was incubated for an additional 48 h. Next cells were fixed, stained with Hoechst 33,342, and imaged using the Opera Phenix Plus (Revvity). The obtained images were analyzed for CRC cell numbers in each well with Harmony software. Cell proliferation was assessed based on the number of CRC cells in co-culture relative to the number of cells in the control wells. Three independent experiments were conducted, with three wells and no less than 4 imaging fields analyzed per experiment.

### Spontaneous migration analysis

To measure migration distance and speed, CRC cells, after indirect co-culture were seeded in 96-well plates (ImageLock, Sartorius). Phase-contrast time-lapse images were captured every 2 h for 24 h using a 10 × objective in an IncuCyte® Live-Cell Analysis System. Cell movement was tracked in ImageJ (Manual Tracking plugin (Schneider et al. 2012)). Migration rate was determined as the mean cell velocity, while the total distance covered by each cell as cumulative track lengths. Three independent experiments were performed, with at least 25 cells analyzed per experiment from three different wells of technical repetition.

For direct co-culture, ASCs were seeded into the wells of a 96-well PhenoPlate (Revvity) and incubated for 24 h. Next, CRC cells, previously stained with the Cell Tracking Red Dye Kit (Abcam), were seeded onto ASCs or into empty wells (control cells). For the 3D migration assay, ASCs and CRC cells were embedded between two layers of Matrigel. Plates were imaged every 2 h for 30 h using the Opera Phenix Plus high-content imaging system. Cell migration was analyzed in Harmony software. Four independent experiments were performed, analyzing at least 400 cells each.

### Collective migration assay

CRC cells were seeded into Matrigel-coated ImageLock 96-well plates and incubated for 24 h. Standardized scratches were made using the Wound Maker™ (Bioscience, Waltham, Massachusetts, USA), followed by overlaying cells with Matrigel for 3D analysis. After gel solidification, a 1:1 mix of fresh and conditioned medium was added. Imaging was conducted as in the spontaneous migration assay, and data were analyzed using the IncuCyte® scratch wound module. Relative wound density reflected cell-covered area over time. Experiments were done in triplicate with 3–4 technical replicates per condition.

### qRT-PCR analysis

RNA was isolated using GeneMATRIX Universal RNA Purification Kit (EURx) including on-column digestion with DNase I (EURx, Gdańsk, Poland), according to the manufacturer’s instructions. Next, the High-Capacity cDNA Reverse Transcription Kit (Applied Biosystems) for reverse transcription was used, following the producer’s guidelines. Quantitative PCR experiments were performed using PowerUp™ SYBR™ Green Master Mix, with normalization to the hypoxanthine phosphoribosyltransferase 1 (HPRT1) gene based on the comparative CT method. All primers used in this study were from Merck (Darmstadt, Germany), and their sequences are provided in Table S1 in supplementary material. At least three independent experiments were conducted.

### Confocal microscopy

Confocal microscopy was used to assess the morphology of CRC cells cultured on coverslips and fixed with 4% formaldehyde. Next, cells were washed and subjected for cytoplasm staining with CellMask deep red (Invitrogen, Waltham, Massachusetts, USA). Coverslips were mounted on slides using Dako mounting medium (Agilent Technologies, Santa Clara, CA, USA). Further observations were performed with a confocal scanning microscope (Leica SP8; LasX 3.3.0 software and the Opera Phenix Plus (Revvity) Harmony) with water objective 40 × or 20x. At least three independent experiments were conducted with no less than 3 imaging field analyzed. Quantification of spindle-like shaped cells percentage was performed with Opera Phenix Plus microscope and Harmony software. Cells were qualified as elongated when they not exceed 0.38 width-length ratio. Quantification of roundness of elongated cells in direct co-culture in relation to cells in monoculture was performed with ImageJ software. At least three independent experiments were conducted with no less than 20 cells measured for each repetition and condition.

### Western blotting analysis

Protein lysates and Western blotting were performed according to the previously described procedure (Olszańska et al. 2023). Utilized antibodies, mouse: anti-STAT3 (Santa Cruz Biotechnology, Dallas, CA, USA), anti-E-cadherin (Cell Signaling Technology, Danvers, MA, USA); rabbit: anti-ERK1/2 (Cell Signaling), anti-p38 (Cell Signaling), anti-EGFR (Cell Signaling). At least three independent experiments were conducted.

### Spheroid formation and characterization

Spheroids were formed using the standard hanging droplet protocol (Foty 2011). Cells were seeded in droplets on the lid of a culture dish. Spheroids were allowed to form for seven days before being subjected to further analysis. Spheroid shape and size were evaluated with phase contrast microscope (Leica DMI3000) after transferring the spheroids to a 24-well plate. Further analysis were conducted using ImageJ software. Images of forming spheroids were captured under a binocular microscope Stemi305 (Zeiss) while still in the hanging droplet. At least three independent experiments were conducted with 4–10 spheroids per repetition.

### Spheroid migration assay

Cell migration from the spheroid was assessed for the formed spheroids placed on the 24-well plate in fresh medium. The spreading of formed spheroids were imaged over time for 7 days with phase contrast microscope (Leica DMI3000). Further analysis was conducted using ImageJ software. Migration intensity of cells from spheroid was quantified by measuring the maximum distance from the spheroid's edge to the center along the migration axis for at least three independent experiments with 4–10 spheroids per repetition.

### Statistical analysis

Data are presented as mean ± standard deviation (SD) with 95% confidence intervals (CI), derived from at least three biologically independent replicates. Group comparisons were performed using two-way ANOVA with Tukey’s multiple comparisons post-hoc test. Statistical significance was considered at *p* < 0.05 (*), *p* < 0.01 (**), *p* < 0.001 (***), and *p* ≤ 0.0001 (****). All analyses were conducted using GraphPad Prism 9 (GraphPad Software, San Diego, CA, USA).

## Results

**Fig. 2:**
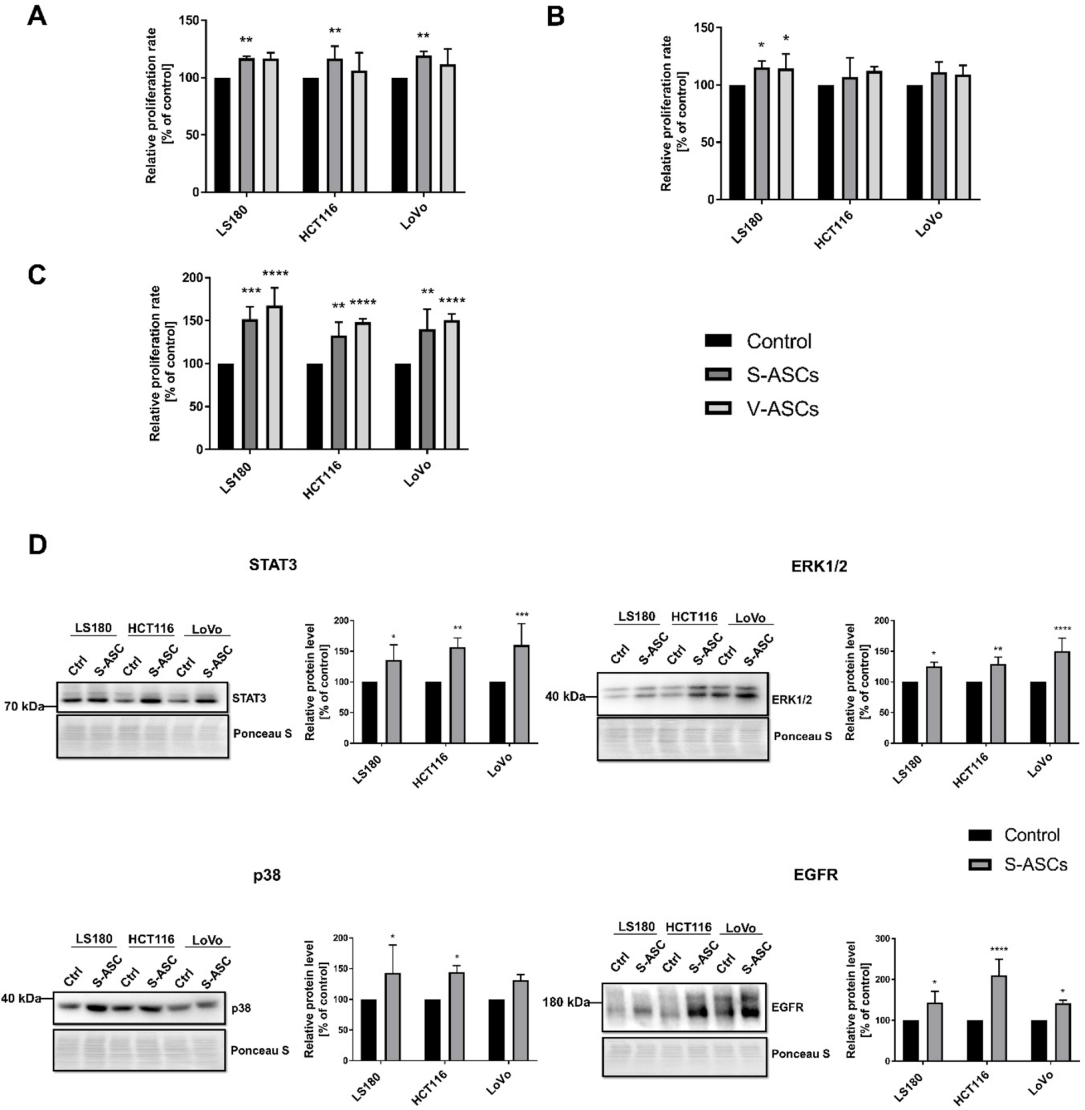
Proliferation of CRC cells in co-culture with ASCs. The proliferation rate of CRC cells following indirect Transwell co-culture with S-ASCs and V-ASCs was measured at 24 h applying (**A**) video-microscopy and (**B**) the XTT assay. **C** Proliferation rate of CRC cells in direct co-culture with S-ASCs and V-ASCs evaluated with video-microscopy. **D** Western blotting analysis of the levels of factors related to cell proliferation and migration abilities in indirect co-culture: STAT3, ERK1/2, p38, and EGFR. The total protein content assessed by Ponceau S staining was used for normalization of Western Blotting analysis. Analyses were performed in three to five independent experiments (*n* = 3–5). Data are presented as mean ± SD of relative values normalized to control cells. Statistical analysis was performed using two-way ANOVA followed by Tukey’s multiple comparison test. Significance levels are indicated as: *p* ≤ 0.05 (*), *p* ≤ 0.01 (**), *p* ≤ 0.001 (***) or *p* ≤ 0.0001 (****)

The TME plays a vital role in the progression of cancer, and the intensive proliferation of tumor cells is one of the crucial factors. Therefore, we investigated whether the presence of ASCs within the tumor niche affects this feature. Using an indirect model, we observed a slight increase in the proliferation rate of CRC cells that were co-cultured with S-ASCs (Fig. 2A) or V-ASCs (Fig. 2B). Interestingly, when applying the direct co-culture model (Fig. 2C), there was a substantial acceleration in the proliferation of cancer cells. To explore the molecules responsible for the pro-proliferative effect, we analyzed the expression levels of STAT3, ERK1/2, EGFR, and p38 proteins (Fig. 2D). The results indicated increased levels of these proliferation-regulating proteins in CRC cells upon indirect co-culture, which may explain the changes observed in the CRC-cell behavior. Although V-ASCs are likely to have more active involvement in CRC progression, S-ASCs were used in some experiments due to their broader characterization and reproducibility.

**Fig. 3:**
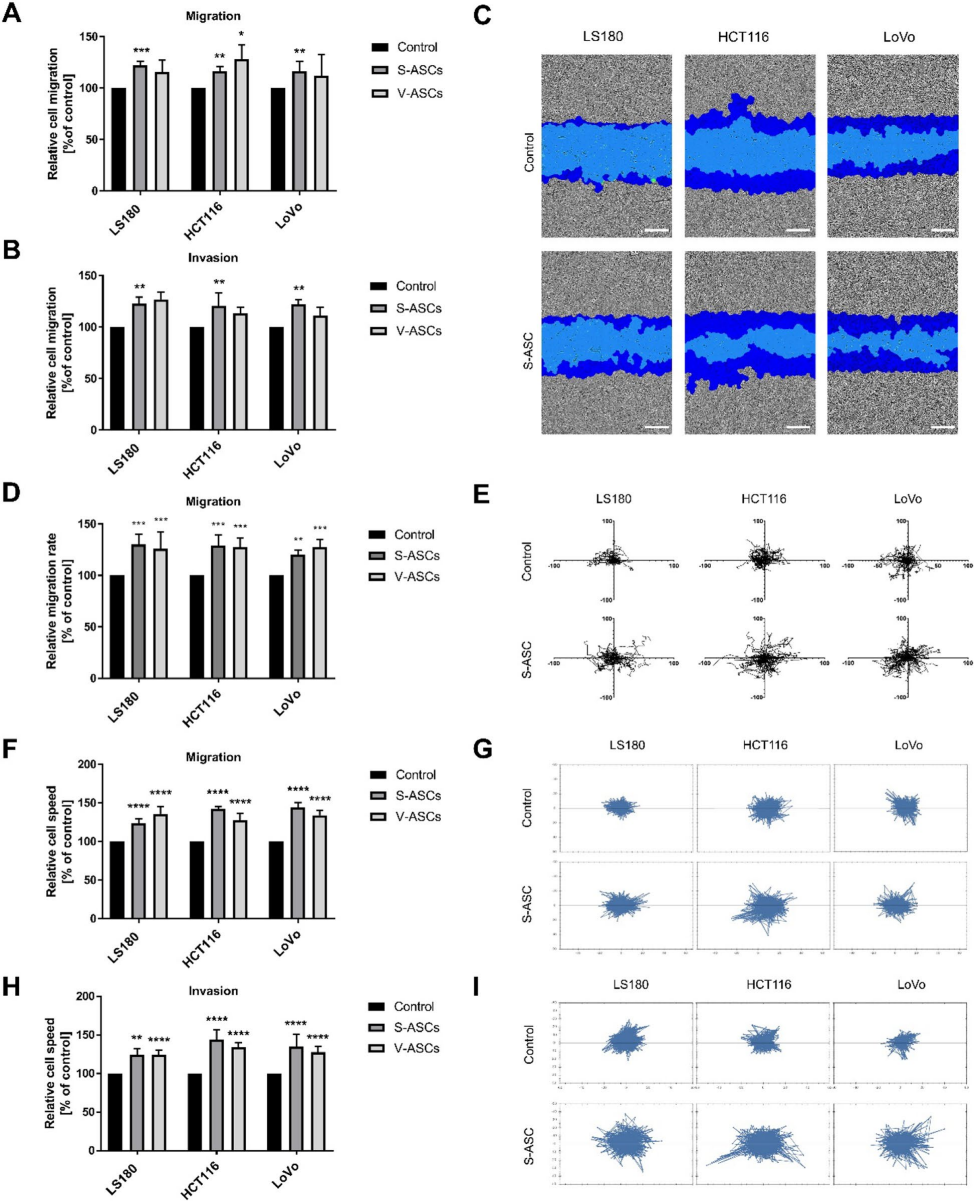
Migratory and invasive potential of CRC cells in co-culture with ASCs. Collective migration ability of CRC cells after indirect co-culture under (**A**) 2D and (**B**) 3D conditions, analyzed after 24 h with the IncuCyte Zoom system. **C** Representative images of the wound after 24 h of migration in 2D conditions, with the wound area marked in light blue and the distance migrated by cells in dark blue (scale bar—300 µm) (**D**, **E**). The spontaneous migration ability of CRC cells was analyzed using the IncuCyte Zoom system for CRC cells after indirect co-culture. **D** Represents cell speed for 24 h period and (**E**) spider plots illustrating cell migration trajectories. A minimum of 25 cells were quantified per experimental repetition. **F**-**I** spontaneous migration of CRC cells in direct co-culture under 2D and 3D conditions. **F** Relative cell speed measurements for cell migration in 2D, with (**G**) spider graphs visualizing migration trajectories of tracked cells. **H** 3D conditions analysis of CRC cell speed in direct co-culture with S-ASCs and (**I**) migration trajectories spider graph. Direct migration/invasion experiments were conducted using the OperaPhenix Plus system with counting of more than 500 cells for repetition. Analyses were performed in three to five independent experiments (*n* = 3–5). Data are presented as mean ± SD of relative values normalized to control cells. Statistical analysis was performed using two-way ANOVA followed by Tukey’s multiple comparison test. Significance levels are indicated as: *p* ≤ 0.05 (*), *p* ≤ 0.01 (**), *p* ≤ 0.001 (***) or *p* ≤ 0.0001 (****)

Recognizing that the investigated signaling molecules are also linked to migration abilities, we analyzed the motility of CRC cells, which is another crucial feature that is inherently linked to the aggressiveness of tumors. In the indirect co-culture model, we observed an increase in collective migration in both 2D conditions (Figs. 3A and C) and 3D conditions (Fig. 3B). These findings motivated us to evaluate the migration of a single cell to further measure the effect of ASCs on CRC cells’ migratory potential. The results showed that following indirect co-culture with S-ASCs and V-ASCs, CRC cells exhibited increased spontaneous motility (Figs. 3D and 3E).

Given the significant role of direct cell–cell contact observed in proliferation experiments, we measured cancer-cell migration in a direct co-culture model. The CRC cells exhibited increased cell speed during co-culture (Fig. 3F) and broader cell trajectories (Fig. 3G) in 2D conditions. A similar effect was observed in 3D conditions, with accelerated cell speed (Fig. 3H) and more dynamic migration patterns, which are illustrated in spider graphs (Fig. 3I). These processes may be triggered by molecules secreted by ASCs. Interestingly, we identified increased production of proteins such as insulin-like growth factor binding protein 3 (IGFBP3), angiogenin (ANG), and vascular endothelial growth factor (VEGF) by S-ASCs (Figure S2).

**Fig. 4:**
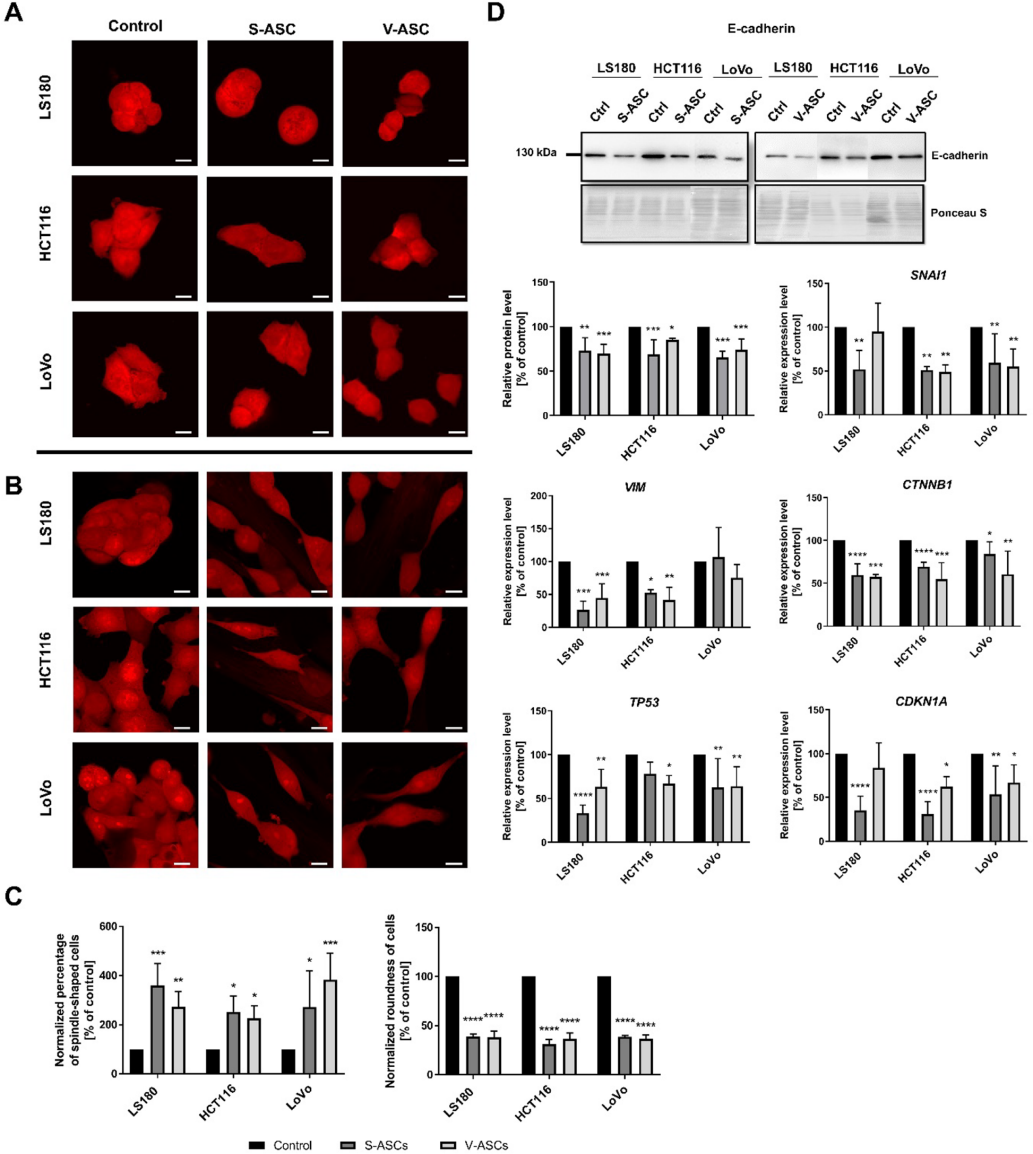
Analysis of CRC cell morphology and expression levels of markers associated with mesenchymal and epithelial phenotypes. Representative images of LS180, HCT116 and LoVo cells upon (**A**) indirect and (**B**) direct co-culture with S-ASCs and V-ASCs. Cells were stained with CellMask Deep to visualize cell shape (scale bar—37 µm). **C** Percentage of cells with spindle-like morphology and roundness of cells in monoculture and of elongated cells in direct co-culture. **D** The level of E-cadherin in CRC cells following indirect co-culture with S-ASCs and V-ASCs was analyzed by Western blotting normalized to total protein amount estimated with Ponceau S staining (explanation of figure assembly – Figure S3). The expression of *VIM* (*vimentin*), *snail family transcriptional repressor 1 (SNAI1)*, *β-catenin* (*CTNNB1*), *CDKN1A* (*p21*) and *TP53* (*p53*) by cells following indirect co-culture with S-ASCs and V-ASCs was analyzed using qRT-PCR normalized to the expression of *HPRT1*. Analyses were performed in three to four independent experiments (*n* = 3–4). Data are presented as mean ± SD of relative values normalized to control cells. Statistical analysis was performed using two-way ANOVA followed by Tukey’s multiple comparison test. Significance levels are indicated as: *p* ≤ 0.05 (*), *p* ≤ 0.01 (**), *p* ≤ 0.001 (***) or *p* ≤ 0.0001 (****)

Recent studies have highlighted the role of V-ASCs in promoting the metastatic potential of CRC sphere cells (Di Franco et al. 2021) and inducing EMT of CRC cells through conditioned medium collected from ASCs (Wei et al. 2015). This inspired us to investigate whether indirect co-culture with ASCs form different fat deposits could affect EMT in CRC cells. One hallmark of mesenchymal phenotype acquisition is a change in cell morphology, so we first assessed the shape of CRC cells. We did not observe any changes in cell morphology after indirect co-culture (Fig. 4A). However, CRC cells in direct co-culture with both S-ASCs and V-ASCs exhibited a spindle-like shape (Fig. 4B). To validate this observation, we quantified the percentage of cells exhibiting spindle-like morphology in monoculture and in direct co-culture with S-ASCs and V-ASCs. A significantly higher proportion of elongated cells was observed in direct co-culture conditions than in monoculture (Fig. 4C). To assess the extent of morphological changes, we measured the roundness of cells in monoculture and of elongated cells in direct co-culture (Fig. 4C). A significant decrease in cell roundness was detected in co-cultured cells, indicating a pronounced elongation phenotype.

We evaluated the expression of E-cadherin to investigate whether these changes are associated with the EMT process and whether they result from the secretion of soluble factors by ASCs. E-cadherin is a key epithelial marker in indirect co-culture. We observed a significant reduction in E-cadherin level in CRC cells that were indirectly co-cultured with both types of ASCs (Fig. 4D). To further analyze the EMT phenotype, we examined the expression levels of *vimentin*, *snail family transcriptional repressor 1 (SNAI1),* and *β-catenin (CTNNB1)*, which were all reduced after indirect co-culture (Fig. 4D). Additionally, we analyzed the expression of *cyclin dependent kinase inhibitor 1 A (p21)* and *tumor protein 53 (p53)*, which are known to modulate EMT process. The results showed that they were both downregulated (Fig. 4D).

**Fig. 5:**
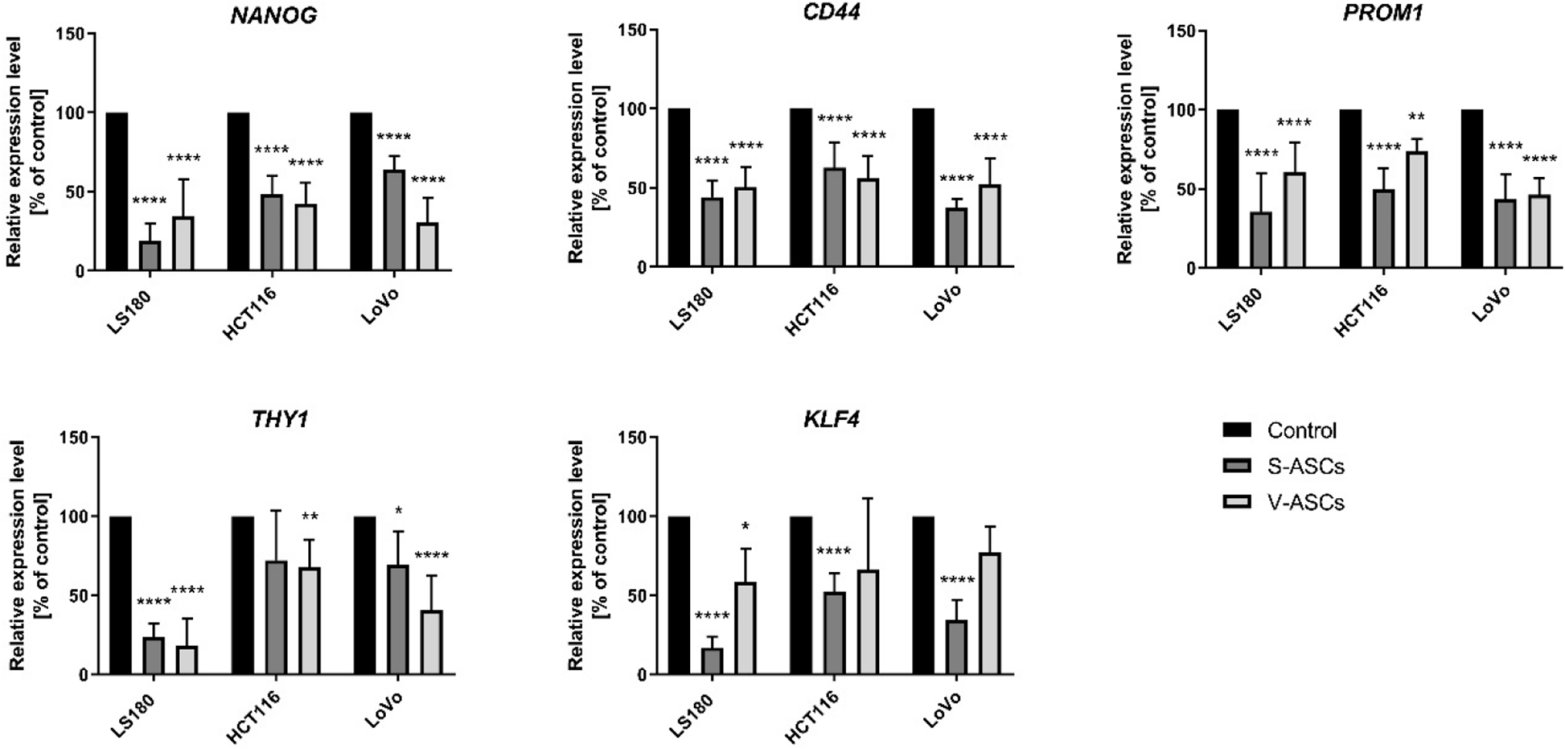
Stemness-related markers in CRC cells after co-culture with ASCs. The expression levels of stemness-associated markers: *nanog homeobox* (*NANOG*), *CD44*, *prominin 1* (*PROM1; CD133*), *thy-1 cell surface antigen* (*THY1; CD90*), and *kruppel-like factor 4* (*KLF4*) were quantified using qRT-PCR normalized to the expression of *HPRT1*. Measurements were performed for CRC cells upon indirect Transwell co-culture with S-ASCs and V-ASCs. Analyses were performed in three independent experiments (*n* = 3). Data are presented as mean ± SD of relative values normalized to control cells. Statistical analysis was performed using two-way ANOVA followed by Tukey’s multiple comparison test. Significance levels are indicated as: ≤ 0.05 (*), *p* ≤ 0.01 (**), *p* ≤ 0.001 (***)or *p* ≤ 0.0001 (****)

We assessed the stemness cancer cells after indirect co-culture with ASCs. This key factor in tumor development is closely linked to interactions within the TME and cancer aggressiveness. The results indicated downregulation of all tested stemness markers, including *nanog homeobox (NANOG), CD44, prominin 1 (PROM1; CD133), thy-1 cell surface antigen (THY1; CD90),* and *kruppel-like factor 4 (KLF4)* (Fig. 5).

**Fig. 6:**
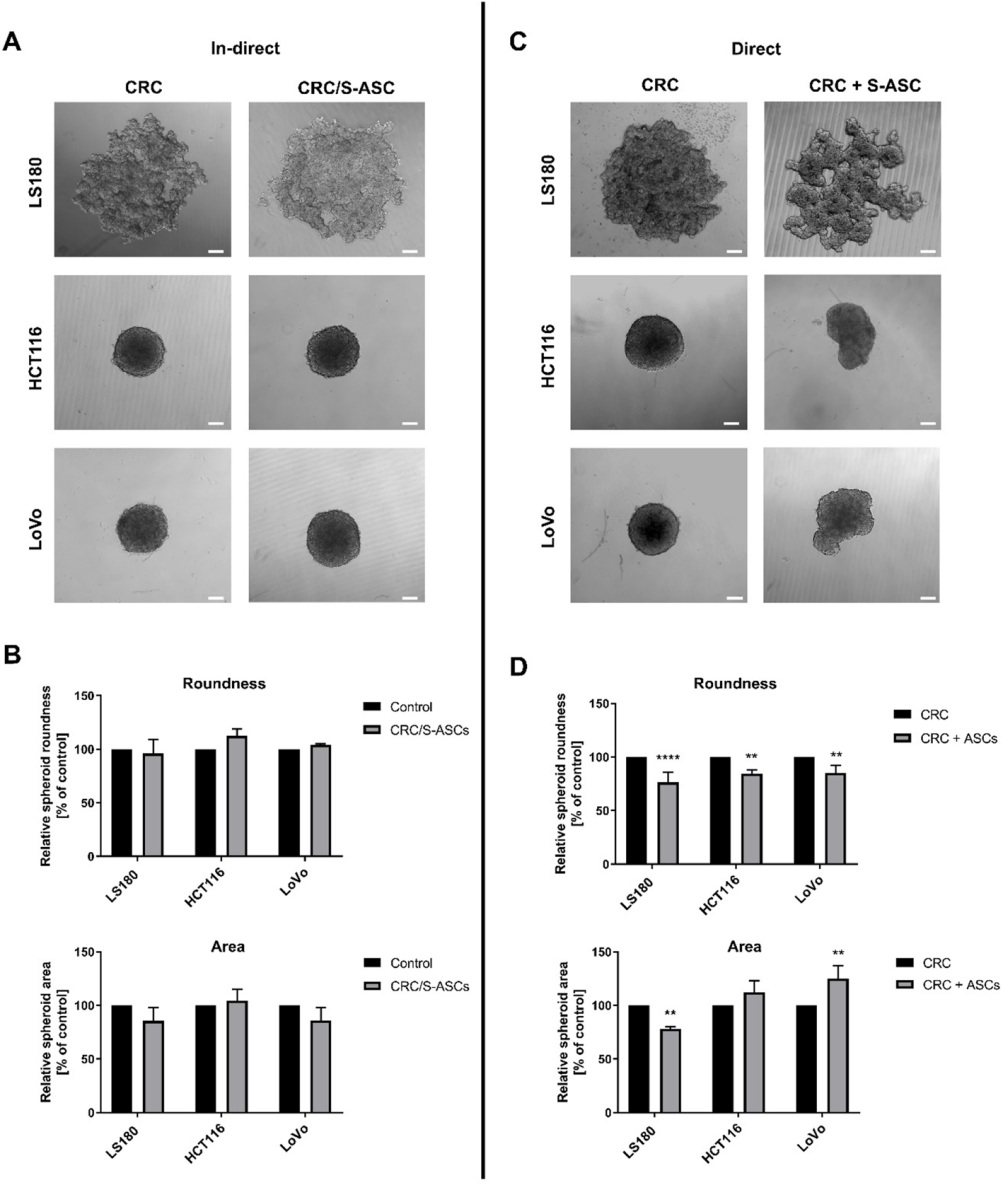
Examination of CRC-derived spheroid morphology upon co-culture. Representative images of spheroids formed by (**A**) control and CRC cells after co-culture with S-ASCs (CRC/S-ASC) and (**C**) control and CRC cells cultured simultaneously with S-ASCs (CRC + ASC; scale bar—150 µm). **B**, **D** Roundness and area of spheroids, following indirect co-culture and during direct co-culture with S-ASCs, respectively. Analysis were performed in three to four independent experiments (*n* = 3–4), with 4–10 spheroids analyzed per experiment. Data are presented as mean ± SD of relative values normalized to control cells. Statistical analysis was performed using two-way ANOVA followed by Tukey’s multiple comparison test. Significance levels are indicated as: *p* ≤ 0.05 (*), *p* ≤ 0.01 (**), *p* ≤ 0.001 (***)or *p* ≤ 0.0001 (****)

The plasticity of the EMT state and the expression of cancer stemness markers are indispensably linked to the ability of CRC cells to form spheroids, which is a hallmark of the cancer stem-like phenotype (Ponomarev et al. 2023; Ishiguro et al. 2017). Therefore, we evaluated whether co-culture with ASCs affects the spheroid-forming capacity of CRC cells. We did not observe visible differences in the spheroid morphology (Fig. 6A), roundness, or area (Fig. 6B) formed by CRC cells upon indirect co-culture with ASCs.

Considering that previous results were more pronounced in the direct co-culture model, we next assessed whether direct interaction with S-ASCs affects the spheroid-forming capacity. To this end, we created mixed spheroids comprising S-ASCs and CRC cells. There were visible changes in the spheroid morphology characterized by loss of spherical structure definition and the emergence of denser formations within the spheroid (Fig. 6C). These observations were confirmed by a significant decrease in spheroid roundness and ambiguous alterations in spheroid area (Fig. 6D).

**Fig. 7:**
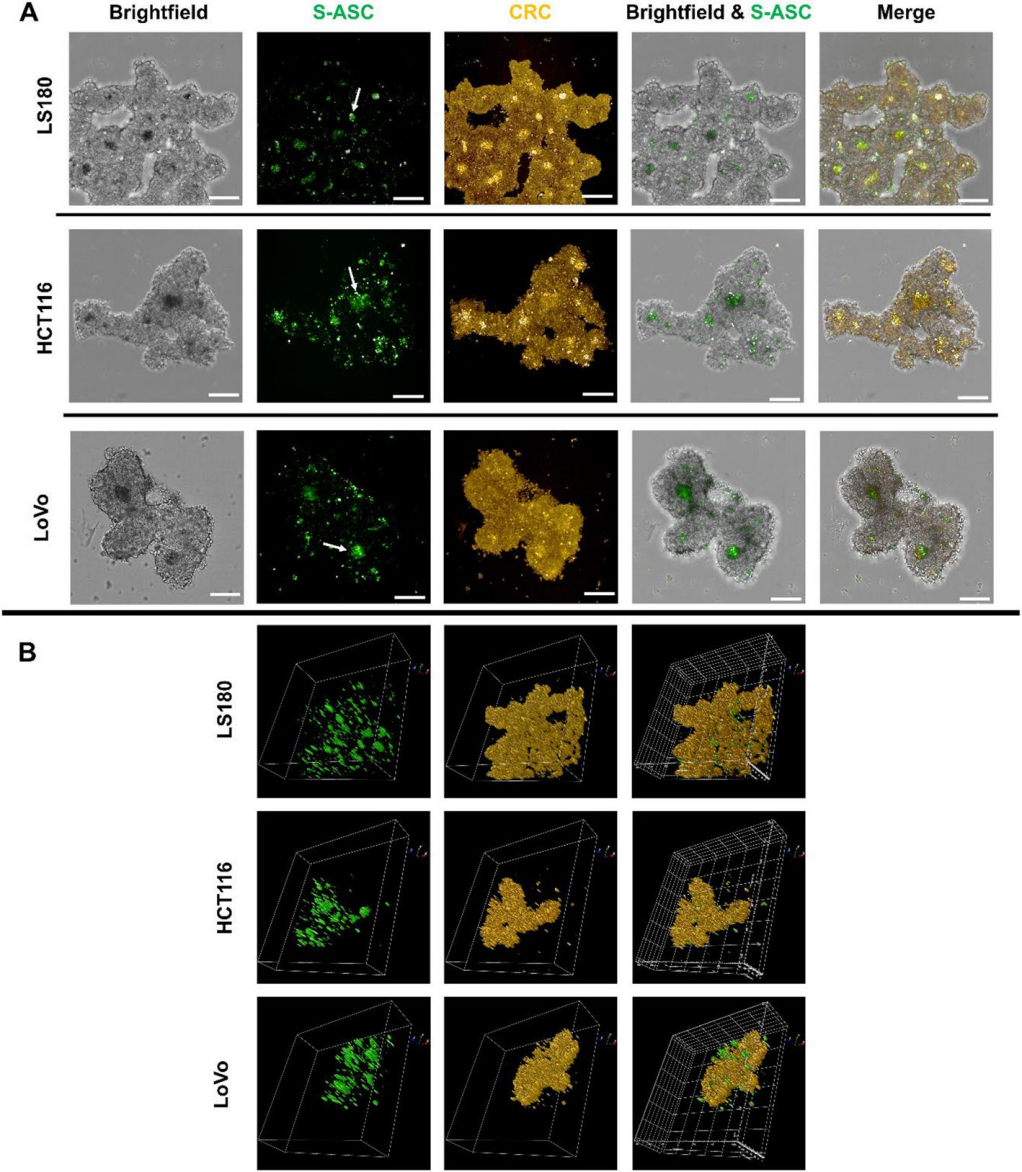
Localization of cells within mixed spheroids formed by CRC and ASCs. Images showing the localization of CRC cells and S-ASCs in mixed spheroids. Different cell types were visualized using specific live-cell dyes, with excitation at 568 nm for CRC cells (yellow) and 488 nm for S-ASCs (green). Formation of ASCs clusters indicate arrows at 488 channel. Spheroid morphology is presented in (**A**) 2D and (**B**) 3D planes (scale bar—200 µm). The 3D visualizations cover a Z-stack of at least 200 µm of the spheroid. Analysis were performed in three to four independent experiments (*n* = 3–4), with 5–9 spheroids analyzed per experiment

The observed alterations in spheroid morphology in direct co-culture motivated us to investigate the spatial localization of cells within mixed spheroids. To achieve this, we fluorescently labeled CRC cells and S-ASCs to visualize their localization after spheroid formation (Fig. 7A). Rather than a single central core, brightfield images showed darkened spheroid centers with higher cell density, which coincided with the localization of S-ASCs (green). CRC cells (yellow) were evenly distributed throughout the spheroid, yet they tended to form more compact structures in the vicinity of these S-ASCs centers. 3D imaging analysis (Fig. 7B) performed on spheroids with a thickness of at least 200 µm confirmed that cells were distributed throughout the entire structure.

**Fig. 8:**
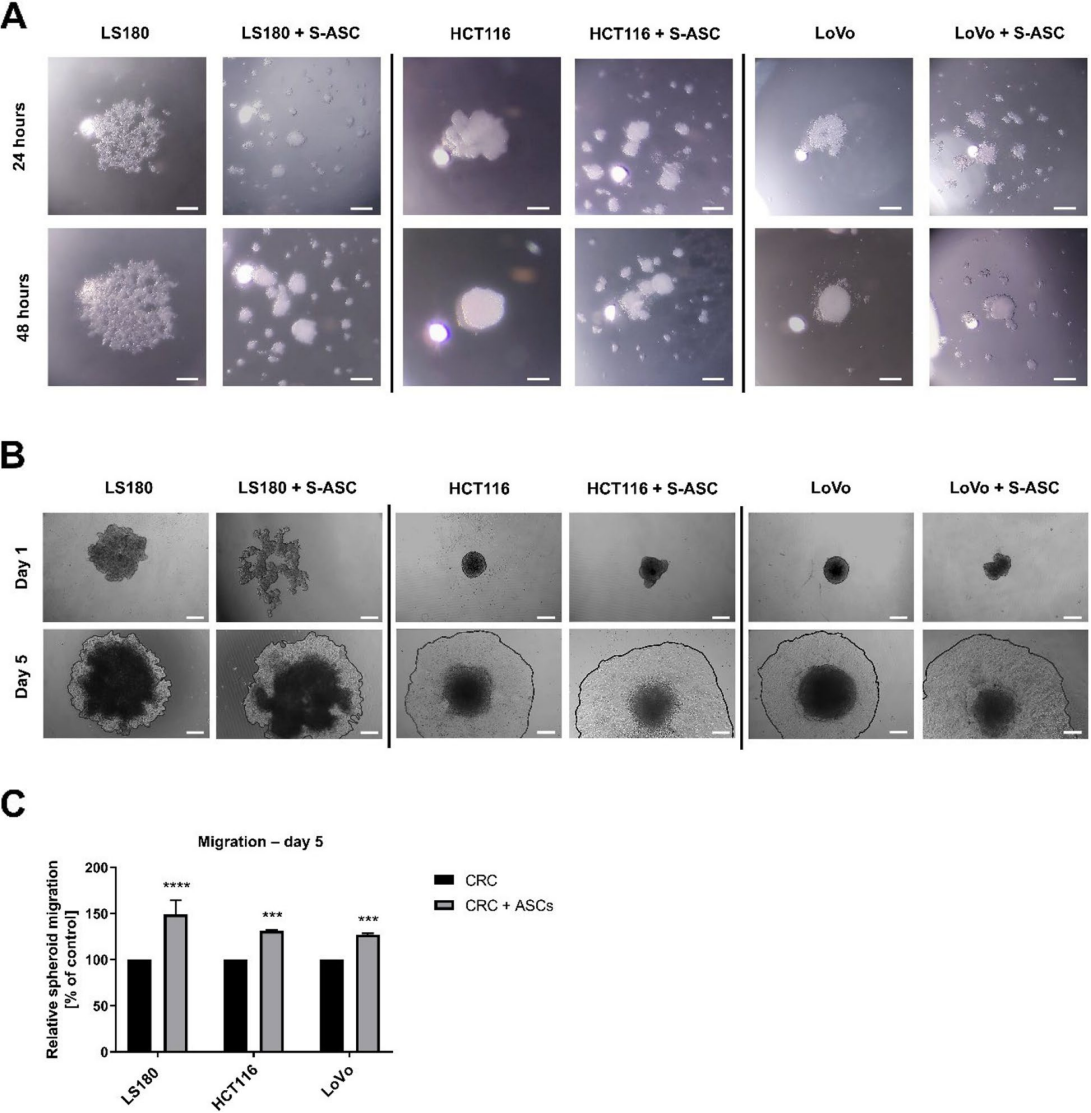
Spheroid formation and migration assay of mixed spheroids formed by CRC cells and ASCs. **A** Representative images showing the formation (in hanging-drop) of spheroids formed by 1 or 2 types of cells after 24 and 48 h following droplet placement (scale bar—160 µm). **B** Images of migration of cells from spheroids created by CRC and S-ASCs 1 and 5 days following placement on the plate (scale bar—300 µm). **C** Quantitative analysis of the migration distances at day 5. Analysis were performed in three to four independent experiments (*n* = 3–4), with 4–10 spheroids analyzed per experiment. Data are presented as mean ± SD of relative values normalized to control cells. Statistical analysis was performed using two-way ANOVA followed by Tukey’s multiple comparison test. Significance levels are indicated as: *p* ≤ 0.01 (**), *p* ≤ 0.0001 (****)

Based on the differences in spheroid characteristics and specific cellular distribution, we sought to determine whether these changes occur during the initial stages of spheroid formation or result from later cell migration within the spheroid. We monitored spheroid formation in a hanging-droplet and captured images at 24 and 48 h after droplet placement (Fig. 8A). Control CRC cells aggregated at the bottom of the droplet and formed a compact and rather homogenous structure. In contrast, co-culture spheroids formed multiple cell clusters that were smaller and denser at the early stage of formation. A typical feature of spheroids is also the ability to disperse and spread. Considering the previously noted increase of CRC cell migration in direct co-culture, we next evaluated cell migration from mixed spheroids. We observed a significant increase in cell migration from mixed spheroids compared to the control spheroids, as shown in representative images in Fig. 8B and the distance migrated by cells measured from the spheroid center in Fig. 8C. We did not observe analogous differences for spheroids from CRC cells upon indirect co-culture (Figure S1).

## Discussion

The vast majority of current related research indicates that adipocytes and ASCs in the TME secrete various factors that promote tumor growth and metastasis (Domagalski et al. 2025; Nimri et al. 2019; Pham et al. 2023). However, the findings are mainly based on Transwell co-culture systems or conditioned media, which focus on secreted factors while neglecting the role of direct cell–cell interactions (Xue et al. 2022a, 2022b; Ko et al. 2019). While some studies employ mouse models that better mimic the TME (Xue et al. 2022b; Wang et al. 2024; Yoon et al. 2021), they are not fully representative of human tumors, and their complexity makes it difficult to pinpoint the cellular components that have the greatest effect on cancer cells. These issues raise questions about whether direct contact between ASCs and CRC cells is a critical element that promotes tumor progression.

Recent studies indicate that ASCs can promote tumor growth and metastasis by stimulating the proliferation and migration of cancer cells (Xue et al. 2022b; Preisner et al. 2018; Wang et al. 2017). The present results demonstrate that ASCs increase CRC cell proliferation and promote both collective and single-cell migration in indirect co-culture, which is consistent with previous findings (Wei et al. 2015; Di Franco and Stassi 2021; Zhang et al. 2018; Chen et al. 2015). Interestingly, the present data highlight the importance of direct cell–cell contact within the TME as the promoting effect was more pronounced in the direct co-culture model, even after a shorter co-culture period.​ A similar pro-proliferative effect has also been reported for ASCs isolated from mouse adipose tissue in both direct and indirect co-culture systems, which aligns with the present findings for human ASCs (Wei et al. 2015; Trivanović et al. 2014), and a similar effect has been observed for other types of cancer (Trivanović et al. 2014).

Additionally, we observed increased migration of CRC cells during the course of co-culture, even after a short period in both2D and 3D conditions Increased migration and proliferation have been linked to activation of the STAT3-dependent pathway (Wei et al. 2015; Wang et al. 2017; Zhang et al. 2018), although this activation may vary with the co-culture duration, which is a critical factor in TME models (Zhang et al. 2018; Zhu et al. 2014). With long-term co-culture, upregulation of STAT3, ERK1/2, p38, and EGFR occurred, which suggests that early molecular shifts correlate with the observed proliferation and migration increases and may be associated with the more pronounced effects in direct co-culture.

Although the data reveal consistent phenotypic and signaling changes associated with co-culture, there is a limitation to the study in regard to the confirmation of the molecular mechanisms with blocking or rescue experiments. Thus, the observed modulation of the mentioned pathways should be interpreted as associative rather than confirmed drivers of the phenotype. Interestingly, some research indicates that the secretome of ASCs may inhibit the growth of CRC cells and trigger their apoptosis (Khodayar et al. 2023). Our research has identified ASC-derived factors that may underlie the observed effects—notably IGFBP3, ANG, and VEGF, which are implicated in processes such as cell migration, proliferation, survival, EMT, and angiogenesis (Navarro et al. 2020; Li et al. 2019; Bhattacharya et al. 2017).

The results indicate that co-culture with ASCs may influence EMT-like phenotypic dynamics in CRC cells. In direct co-culture, we observed a morphological shift toward an elongated spindle-like shape that may reflect the initial loss of epithelial characteristics (Ribatti et al. 2020). Interestingly, in the case of indirect co‑culture, molecular inconsistency of the EMT phenotype began to emerge, as shown by a reduction in the levels of E-cadherin, a key epithelial marker (Zhu et al. 2021). However, further analysis of additional EMT-related markers such as vimentin and SNAI1 did not support a complete reprogramming to a fully mesenchymal phenotype (Ribatti et al. 2020). The differences in expression of canonical EMT markers and the decreased p53 and p21 expression observed in the indirect co-culture may reflect transcriptional changes that resemble features of non-classical EMT/MET-related plasticity (Pastushenko and Blanpain 2019).

This discrepancy suggests that the observed alterations may reflect a stress response or some kind of morphological adaptation rather than a full canonical EMT. Accordingly, we describe the phenotype as exhibiting EMT-related features based primarily on transcriptional and morphological tendencies and emphasize that multiple protein-level mesenchymal induction was not observed (Pastushenko and Blanpain 2019; Nieto et al. 2016). These findings highlight the complexity of interpreting EMT-like processes in this context and underscore the need for future studies to confirm the functional significance.

Downregulation of both epithelial and mesenchymal markers has been described in tumor–stroma crosstalk and reflect a shift toward intermediate states in which cells exhibit reduced EMT marker expression while retaining functional migratory capacity (Pastushenko and Blanpain 2019; Bodmer et al. 2025; Fontana et al. 2025). Moreover, suppression of p53 and p21 may further contribute to this phenotype. Loss of p53 can disrupt epithelial identity by downregulating E-cadherin as this molecule has been shown to promote CDH1 transcription and maintain its expression (Chang et al. 2011; Semenov et al. 2022). Additionally, p53 deficiency can perturb Wnt/β-catenin signaling, which regulates epithelial differentiation programs (Shi et al. 2020; Xiao et al. 2022). Interestingly, p21 deficiency may increase cellular plasticity and promote states that are compatible with hybrid or partial EMT phenotypes. This effect is consistent with its role in interacting with ZEB1 and miRNA clusters to inhibit this process, as well as broader studies on EMT plasticity (Bueno-Fortes et al. 2022; Li et al. 2014).

These effects may also be related to changes in the ASC secretome induced by crosstalk with colorectal cancer cells. In this context, adipose-derived stromal cells have been shown to increase the secretion of IL-6, HGF, TGF-β, and CXCL12, which may activate multiple signaling pathways and thereby potentially promote EMT or hybrid/partial EMT states in colorectal cancer cells (Di Franco et al. 2021; Zhu et al. 2024; El Alaa et al. 2024). Interestingly, differences in the secretome of visceral and subcutaneous ASCs may lead to differential modulation of EMT in tumor cells (Ritter et al. 2015; El Alaa et al. 2024). These findings suggest that CRC cells may undergo processes related to stress response or morphological adaptation that are potentially connected with fluctuations in EMT-like plasticity, adopting a “plastic” state, particularly in light of the notable heterogeneity observed in EMT-associated characteristics.

The increase observed in the proliferative and migratory capabilities of CRC cells may be associated with the emergence of EMT-like phenotypic plasticity (Jolly et al. 2015). The observed features were significantly increased in the direct co-culture compared to the indirect co-culture, with morphological changes indicative of mesenchymal characteristics appearing exclusively in direct co-culture conditions. Together with the ambiguous alterations in the molecular EMT-like phenotype of CRC cells in the indirect co-culture, these findings suggest that direct cell–cell interactions may be important in the modulation of CRC cell aggressiveness in the presence of ASCs.

Similar observations regarding the EMT status of CRC sphere cells co-cultured with ASCs have been reported (Di Franco et al. 2021). Furthermore, a recent analysis of CRC patients, patient-derived xenografts, or other cellular models indicate an ambiguous epithelial-mesenchymal phenotype state with transcriptomic profile classified as hybrid, reversible, or partial (George et al. 2017; Jolly et al. 2019; Mizukoshi et al. 2020; Hiew et al. 2018; De Angelis et al. 2022). This underscores the complexity of EMT-related outcomes and suggests a contributory role of microenvironmental cells in shaping this state.

Inconsistency in the EMT phenotype is typically linked to the preservation of cancer cell stemness (Jolly et al. 2019; Sabouni et al. 2023), so we evaluated stem-cell-related markers in CRC cells following co-culture with ASCs. Interestingly, most of the tested markers showed downregulated expression, which is unexpected since it has been implied that plasticity of the EMT state promotes stemness in cancer cells (Jolly et al. 2015; Jolly et al. 2019). Although transcriptional analyses indicate present downregulation in stemness-related markers in ASCs following co-culture, functional assays did not reveal significant differences. This uncoupling highlights the importance of interpreting transcriptional changes cautiously as they may not directly translate into classical functional stemness but could instead reflect a broader process of cancer-cell reprogramming.

However, recent studies suggest a possible uncoupling between EMT and stemness (Nieto et al. 2016). Interestingly, it has been proposed that the interplay between LIN28 and Let-7 can shift the “stemness window” towards different EMT phenotypes, which depends on the activity of their upstream regulators (Jolly et al. 2015). Various EMT states may also be associated with the acquisition of cancer cell stemness, which is influenced by intracellular signaling pathways involving SNAIL, TWIST, or other EMT-related transcription factors (Nieto et al. 2016). Therefore, the present results add another layer of complexity to the understanding of the correlations between EMT and stemness and underscore the role of TME components as critical contributors to these processes.

Interestingly, while ASCs appear to influence certain aspects of CRC cell aggressiveness, such as growth and motility, they do not increase tumor-initiating capacity or stemness functions in indirect co-culture. This functional uncoupling suggests that it may be possible that ASCs selectively modulate specific facets of tumorigenesis without broadly increasing stemness. These observations highlight the complexity of stromal–cancer interactions and indicate that EMT-associated processes and stemness can be uncoupled in certain contexts, as well as the importance of considering both functional and molecular outcomes when interpreting the tumor-promoting effects of ASCs.

Due to the fluctuations in EMT-like phenotypic plasticity and stemness‐related features, we examined the spheroid formation capacity of cancer cells. There were no significant changes in spheroid morphology and size after indirect co-culture, which aligns with the absence of increased expression of stemness markers (Gheytanchi et al. 2021). However, recent reports point to a role of human ASCs in the sphere and tumor-formation capacity of CRC cells (Zhu et al. 2024; Guillaume et al. 2022b). Analysis of co-culture spheroids indicated uneven cellular dispersion, with ASCs predominantly being located in the denser regions and CRC cells being distributed more uniformly. This pattern has also been observed in spheroids containing umbilical-cord mesenchymal stem cells and glioblastoma stem cells (Bajetto et al. 2017).

Notably, even the LS180 cell line, which typically does not form spheroids, developed denser and more defined structures when co-cultured directly with ASCs. This pattern suggests that human ASCs participate in the formation of spheroids from CRC cells. The observations during the first 2 days of co-culture spheroid formation and the cell-localization analyses are consistent with the possibility that ASCs may facilitate the organization of spheroid centers and potentially act as initial aggregation sites around which CRC cells assemble. In addition to this role, mixed spheroids exhibit an increased capacity to disperse that facilitates cell migration from the spheroid over time. This feature may be critical for metastasis, where cancer cells leave the primary tumor and invade surrounding tissues (Tang et al. 2025). These findings suggest that ASCs may contribute to the structural organization of mixed spheroids, actively shape tumor architecture, and support the invasion of CRC cells.

Little is still known about the effect of ASCs isolated from different fat deposits (subcutaneous or visceral) on CRC progression. Considering their differences, this matter may be relevant. Variations have been reported in the effect of adipocytes derived from subcutaneous or visceral deposits on CRC (Nimri et al. 2019; Kim et al. 2021). Some studies also take into account the role of S-ASCs and V-ASCs, but data about their potentially different roles in CRC progression are very limited (Di Franco et al. 2021; Guillaume et al. 2022b).

The findings did not indicate major changes in the modification of cell behavior resulting from the effect of S-ASCs or V-ASCs. However, we identified distinct differences in their impact on the expression of EMT- and stemness-related markers in CRC, which require further investigation concerning differences in types of obesity. Notably, given the multiparametric nature of the co-culture system, the results are based on single, well-characterized donors to ensure the robustness of the conclusions. Nevertheless, future studies including a larger donor cohort will be valuable to validate these findings and assess their generalizability.

## Conclusions

Analysis of the spheroid co-culture model based on human cells, the observed increases in migration and proliferation, and the pronounced morphological changes under direct co-culture provide new insights into the complex interplay within the TME of colon cancer. Modulation of EMT-related features and reduced expression of stemness-associated markers in indirect co-culture suggest a potential role in ASC-mediated modulation of tumor aggressiveness. These shifts may represent an initial layer of ASC-driven remodeling that could potentially contribute to the more pronounced phenotypic changes observed in direct co-culture conditions. Notably, these effects were independent of the fat-depot origin of ASCs. Overall, the findings suggest a potential role for ASCs in modulating CRC-cell phenotypes and provide a foundation for future studies on the molecular pathways involved. Interestingly, the pronounced impact of direct cell–cell contact on CRC cell behavior suggests that physical interactions within the TME may represent an additional regulatory layer alongside paracrine signaling and could be explored in future studies as potential therapeutic targets.

## Data Availability

Data will be made available on request.

## Abbreviations

ANG: Angiogenin
ASC: Adipose stem cell
CD133/PROM1: Prominin 1
CTNNB1: Catenin beta 1 (β-catenin)
CRC: Colorectal cancer
EGFR: Epidermal growth factor receptor
EMT: Epithelial-mesenchymal transition
ERK1/2: Extracellular signal-regulated kinases ½
IGFBP3: Insulin-like growth factor binding protein 3
IL-6: Interleukin 6
IL-8: Interleukin 8
KLF4: Kruppel-like factor 4
MET: Mesenchymal-epithelial transition
NANOG: Nanog homeobox
p21: Cyclin dependent kinase inhibitor 1A
p38: P38 mitogen-activated protein kinase
p53: Tumor protein 53
qRT-PCR: Quantitative reverse transcription polymerase chain reaction
SNAIL: Snail family transcriptional repressor 1
STAT3: Signal transducer and activator of transcription 3
S-ASC: Subcutaneous adipose stem cell
THY1/CD90: Thy-1 cell surface antigen
TME: Tumor microenvironment
TNFα: Tumor necrosis factor α
V-ASC: Visceral adipose stem cell
VEGF: Vascular endothelial growth factor
WB: Western blot
